# Hybrid CMR- and FDG-PET-Imaging Gives New Insights Into the Relationship of Myocardial Metabolic Activity and Fibrosis in Patients With Becker Muscular Dystrophy

**DOI:** 10.3389/fcvm.2022.793972

**Published:** 2022-01-31

**Authors:** Volker Vehof, Florian Büther, Anca Florian, Stefanos Drakos, Bishwas Chamling, Peter Kies, Lars Stegger, Ali Yilmaz

**Affiliations:** ^1^Department of Cardiology I, University Hospital Münster, Münster, Germany; ^2^Department of Nuclear Medicine, University Hospital Münster, Münster, Germany

**Keywords:** CMR, PET, FDG, LGE, muscular dystrophy, cardiomyopathy

## Abstract

**Background:**

Cardiac involvement in patients with Becker muscular dystrophy (BMD) is an important predictor of mortality. The cardiac phenotype of BMD patients is characterized by slowly progressive myocardial fibrosis that starts in the left ventricular (LV) free wall segments and extends into the septal wall during the disease course.

**Purpose:**

Since the reason for this characteristic cardiac phenotype is unknown and comprehensive approaches using e.g. hybrid imaging combining cardiovascular magnetic resonance (CMR) with ^18^F-fluorodeoxyglucose (FDG) positron emission tomography (PET) are limited, the present study addressed this issue by a comprehensive non-invasive imaging approach.

**Methods:**

Hybrid CMR- and FDG-PET-imaging was performed in *N* = 14 patients with BMD on a whole-body Biograph mMR system (Siemens, Erlangen, Germany). The CMR protocol comprised cine- and late-gadolinium-enhancement (LGE)-imaging. Metabolism was assessed with FDG-PET after oral glucose loading to effect myocardial carbohydrate uptake. PET was acquired for 65 min starting with tracer injection. Uptake values from 60 to 65 min p.i. were divided by the area under the blood activity curve and reported as percentages relative to the segment with maximal myocardial FDG uptake.

**Results:**

A characteristic pattern of LGE in the LV lateral wall was observed in 13/14 patients whereas an additional septal LGE pattern was documented in 6/14 patients only. There was one patient without any LGE. Segmental FDG uptake was 88 ± 6% in the LV lateral wall vs. 77 ± 10% in the septal wall (*p* < 0.001). There was an inverse relationship between segmental FDG activity compared to segmental LGE extent (*r* = −0.33, *p* = 0.089). There were *N* = 6 LGE-positive patients with a segmental difference in FDG uptake of >15% in the LV lateral wall compared to the septal wall = ΔFDG-high group (lateral FDG = 91±3% vs. septal FDG = 69±8%; *p* < 0.001) while the remaining *N* = 7 LGE-positive patients showed a segmental difference in FDG uptake of ≤ 15% = ΔFDG-low group (lateral FDG = 85±7% vs. septal FDG = 83 ± 5%; *p* = 0.37). Patients in the ΔFDG-high group showed only a minor difference in the LGE extent between the LV lateral wall vs. septal wall (*p* = 0.09) whereas large differences were observed in the ΔFDG-low group (*p* < 0.004).

**Conclusions:**

Segmental FDG uptake—reflecting myocardial metabolic activity—is higher in the LV free wall of BMD patients—possibly due to a higher segmental work load. However, segmental metabolic activity seems to be dependent on and limited by the respective segmental extent of myocardial fibrosis as depicted by LGE-imaging.

## Introduction

Duchenne and Becker muscular dystrophies (DMD and BMD) are the most frequent X-chromosomal recessive neuromuscular disorders and are caused by mutations in the dystrophin gene. Such mutations can either lead to total absence (DMD) or to structural impairment of fragile dystrophin protein (BMD) (1, 2). Since dystrophin is a central protein in the cell membrane of skeletal as well as cardiac muscle cells, particularly BMD patients do not only suffer from skeletal muscle weakness but also from progressive cardiomyopathy (2, 3). Cardiac involvement in BMD patients is characterized by a non-ischemic pattern of left ventricular (LV) myocardial fibrosis starting in the LV free wall and leading to non-ischemic dilated cardiomyopathy (2, 4–6). Heart failure symptoms and ventricular arrhythmias have become a major cause of morbidity and mortality in MD patients (2, 7, 8).

Cardiovascular magnetic resonance (CMR) imaging has developed into the most relevant clinical imaging technique regarding detection of cardiac involvement and monitoring of cardiac disease progression—not only for BMD patients. It cannot only measure shape and function of the heart, but also reveals even subtle structural changes in the myocardium such as fibrosis. Previously, our group described a characteristic pattern of cardiomyopathy in patients with BMD (5), the potential for predicting adverse cardiac events (7) and the detection of diffuse interstitial fibrosis (9) based on multi-parametric CMR data.

Recently, hybrid positron-emission-tomography (PET) magnetic resonance imaging (MRI) systems have been introduced that offer the unique capability of simultaneous PET and MRI for optimal spatial and temporal co-registration. This simultaneous approach is extremely valuable for BMD patients in order to relate different parameters, hitherto acquired separately, with each other on a patient-to-patient and even region-by-region basis. Thereby, the relevance and association of changes in both metabolic and structural abnormalities can be established. Also, simultaneous imaging obviates the need for a second imaging session, a significant relief for the often severely physically impaired patients.

A comprehensive PET-CMR approach in BMD patients to assess different aspects of myocardial changes at the same time in the same cardiac region may well-reveal new insights into pathophysiology of cardiac disease and has not been attempted so far. In this context, we present the first preliminary data of our ongoing PET-CMR-MD research project.

## Methods

### Study Population

Fourteen patients with BMD were prospectively enrolled within the scope of the ongoing PET-CMR-MD research project that focuses on cardiac phenotyping of patients with BMD with different severity of myocardial involvement. The diagnosis of BMD was confirmed by skeletal muscle biopsy evaluation with immunohistochemical analysis of the dystrophin protein and/or a genetic work-up with screening for dystrophin gene mutation. Cardiological work-up included (amongst others) physical examination and hybrid PET-CMR imaging. Blood samples were obtained for laboratory studies including measurement of total creatine kinase (CK), high-sensitive troponin-T (hsTnT), NT-pro-brain-natriuretic-peptide (NT-proBNP) apart from others. This study was approved by the local ethics committee and the federal office for radiation protection (BfS). Written informed consent was obtained from each patient prior to inclusion to this study.

### CMR Acquisition and Data Analysis

All patients included in this study underwent a simultaneous and comprehensive PET-CMR imaging study. The studies were performed on a 3-T PET-MRI system capable of simultaneous PET and MRI image acquisition (Biograph mMR, Siemens Healthcare). The CMR protocol comprised (amongst others) cine-imaging and late-gadolinium-enhancement (LGE)-imaging, ~10–15 min after a cumulative gadolinium (Gadovist®) dose of 0.10 mmol/kg. Cine-imaging was performed using a steady-state-free-precession (SSFP) sequence in three long-axis slices (four-, three- and two-chamber) and a stack of short-axis slices completely covering the LV.

Image analysis and interpretation were performed using commercially available software (cvi42, Circle Cardiovascular Imaging, Calgary, Alberta, Canada). Ventricular volumes, ejection fraction and LV mass were derived by contouring endo- and epicardial borders on the short-axis cine images and indexed to body surface area. LGE extent was assessed on the short-axis contrast images and an image intensity level ≥3 SD above the mean of remote myocardium was used to define LGE indicative of damaged myocardium as described previously and expressed as percentage of total LV mass. Based on the 17-segment AHA myocardial segmentation model, we defined a LV “septal wall” as segments 2, 3, 8, 9 and 14, and a LV “lateral wall” as segments 5, 6, 11, 12 and 16.

### PET Acquisition and Data Analysis

Metabolism was assessed with FDG-PET after oral glucose loading (50 grams) one hour before tracer injection to effect myocardial carbohydrate uptake, in accordance with clinically established protocols (10). Since diabetes was an exclusion criterion for the study, administration of insulin as recommended in the above guidelines for higher blood glucose levels one hour after glucose loading was not necessary. PET was acquired for 65 min in list-mode, where all events are stored for retrospective sorting into time frames. The tracer (~185 MBq) was injected as a slow bolus (duration ~45 s) starting thirty seconds into the list-mode scan. Uptake values from 60 to 65 min were divided by the area under the blood activity curve and reported as percentages relative to the segment with maximal myocardial FDG uptake.

In accordance with our CMR data analyses, we used the same 17-segment AHA myocardial segmentation model for our PET data analyses and defined a LV “septal wall” as segments 2, 3, 8, 9 and 14, and a LV “lateral wall” as segments 5, 6, 11, 12 and 16. Segmental PET analysis was performed using the Cardiac PET tool of the software package PMOD version 3.703 (PMOD Technologies, Zurich).

### Statistical Analysis

Statistical analysis was performed with SPSS (version 26.0, IBM Corp., Armonk, NY). Normally distributed variables are expressed as mean ± SD. Skewed variables are expressed as median and range. Categorical variables are expressed as frequency with percentage. Student's *t*-test was used for comparison of groups concerning normally distributed variables, while Mann-Whitney U test was used for comparison of non-normally distributed variables. Non-parametric Kruskal–Wallis test was used in case of multiple comparisons of non-normally distributed variables. Fisher's exact test was used to compare non-continuous variables expressed as proportions. Non-parametric Spearman correlations were used for correlation analysis of non-normally distributed variables. A *p*-value < 0.05 was considered statistically significant.

## Results

### Study Population

Patient characteristics as well as major laboratory results are illustrated in Table 1. The median age of the present study group of (male) BMD patients was 33 years (19–59 years) and there were only *N* = 3 (21%) patients with loss of walking ability. Median total CK levels were substantially elevated with 858 U/l (374–3.787 U/l) whereas hsTnT levels were only modestly increased. There was no patient with NT-proBNP elevation in our study group.

**Table 1:** Patient characteristics.

	**BMD (all)**	**BMD** **LGE-positive** **ΔFDG-high**	**BMD LGE-positive ΔFDG-low**	***p*** **-value**
	***N*** **= 14**	***N*** **= 6**	***N*** **= 7**	
Age, years	33 (19–59)	41 (22–55)	39 (19–59)	0.94
BMI, kg/m^2^	20 (18–23)	20 (19–21)	20 (18–22)	0.48
CK total, U/l	857 (374–3,787)	958 (374–3,787)	854 (517–1,540)	0.83
hsTrop-T, ng/l	18 (12–50)	23 (16–38)	17 (13–50)	0.77
NT-proBNP elevation (%)	0%	0%	0%	NS
ACE-inhibitor therapy (%)	29%	33%	29%	NS
ß-blocker therapy (%)	14%	17%	14%	NS
Loss of walking ability (%)	21%	33%	14%	NS

### CMR Findings

Major CMR findings are shown in Table 2. Median LV-EF in all *N* = 14 BMD patients was 55% (46–61%) with *N* = 6 (43%) patients showing an impaired LV systolic function with a LV-EF <55%. Median RV-EF was 57% (51–63%) and there was no patient with an impaired RV systolic function (defined as RV-EF <50%). A characteristic pattern of non-ischemic LGE in at least one LV wall segment was observed in *N* = 13 (93%) patients. There was only one patient without any LGE—who also showed a preserved LV and RV systolic function. Presence of any LGE (mostly small areas) in the septal wall was observed in *N* = 6 (43%) compared to *N* = 13 (93%) patients with mostly extensive LGE in the LV lateral wall.

**Table 2:** CMR parameters.

	**BMD (all)**	**BMD** **LGE-positive** **ΔFDG-high**	**BMD LGE-positive ΔFDG-low**	***p*** **-value**
	***N*** **= 14**	***N*** **= 6**	***N*** **= 7**	
LV-EF, %	55 (46–61)	55 (50–61)	53 (46–60)	0.52
LV-EDV index, ml/m^2^	92 (72–115)	92 (78–99)	95 (72–115)	0.67
LV mass index, g/m^2^	64 (50–75)	64 (53–72)	65 (50–75)	0.67
RV-EF, %	57 (51–63)	57 (51–62)	57 (53–63)	0.94
RV-EDV index, ml/m^2^	85 (72–106)	85 (72–100)	91 (74–106)	0.89
LGE presence, n (%)	13 (93)	6 (100)	7 (100)	1.0
Global LGE extent, %	7 (0–47)	6 (3–19)	17 (4–47)	0.09
Septal LGE extent, %	0 (0–36.8)	0 (0–36.8)	1.4 (0–15.1)	0.72
Lateral LGE extent, %	17.8 (0–74.5)	13.3 (3.1–18.9)	36.3 (9.9–74.5)	**0.018**

*Bold font indicates a p-value < 0.05*.

### FDG-PET Results in Relation to CMR Findings

Segmental FDG uptake was 88 ± 6% in the LV lateral wall vs. 77 ± 10% in the septal wall in the entire study group (*p* < 0.001). In more detail, segmental FDG uptake in those patients with a total LGE extent of ≤ 10% was 89 ± 6% in the lateral wall vs. 75 ± 10% in the septal wall (*p* = 0.011). In contrast, there was no significant difference in segmental FDG uptake in those patients with a total LGE extent of >10% (86 ± 8% in the lateral wall vs. 79 ± 8% in the septal wall, *p* = NS). There was a moderate inverse relationship between segmental FDG activity compared to segmental LGE extent (*r* = −0.33, *p* = 0.089).

There were *N* = 6 LGE-positive patients with a segmental difference in FDG uptake of >15% in the LV lateral wall compared to the septal wall = ΔFDG-high group (lateral FDG = 91 ± 3% vs. septal FDG = 69 ± 8%; *p* < 0.001) while the *N* = 7 LGE-positive patients showed a segmental difference in FDG uptake of ≤ 15% = ΔFDG-low group (lateral FDG = 85 ± 7% vs. septal FDG = 83 ± 5%; *p* = 0.37). There was no significant difference in “global” as well as “septal” LGE extent between the ΔFDG-high vs. ΔFDG-low group (Table 2). In contrast, there was a substantial difference in “lateral” LGE extent with significantly larger areas of LGE in the ΔFDG-low group.

**Figure 1:**
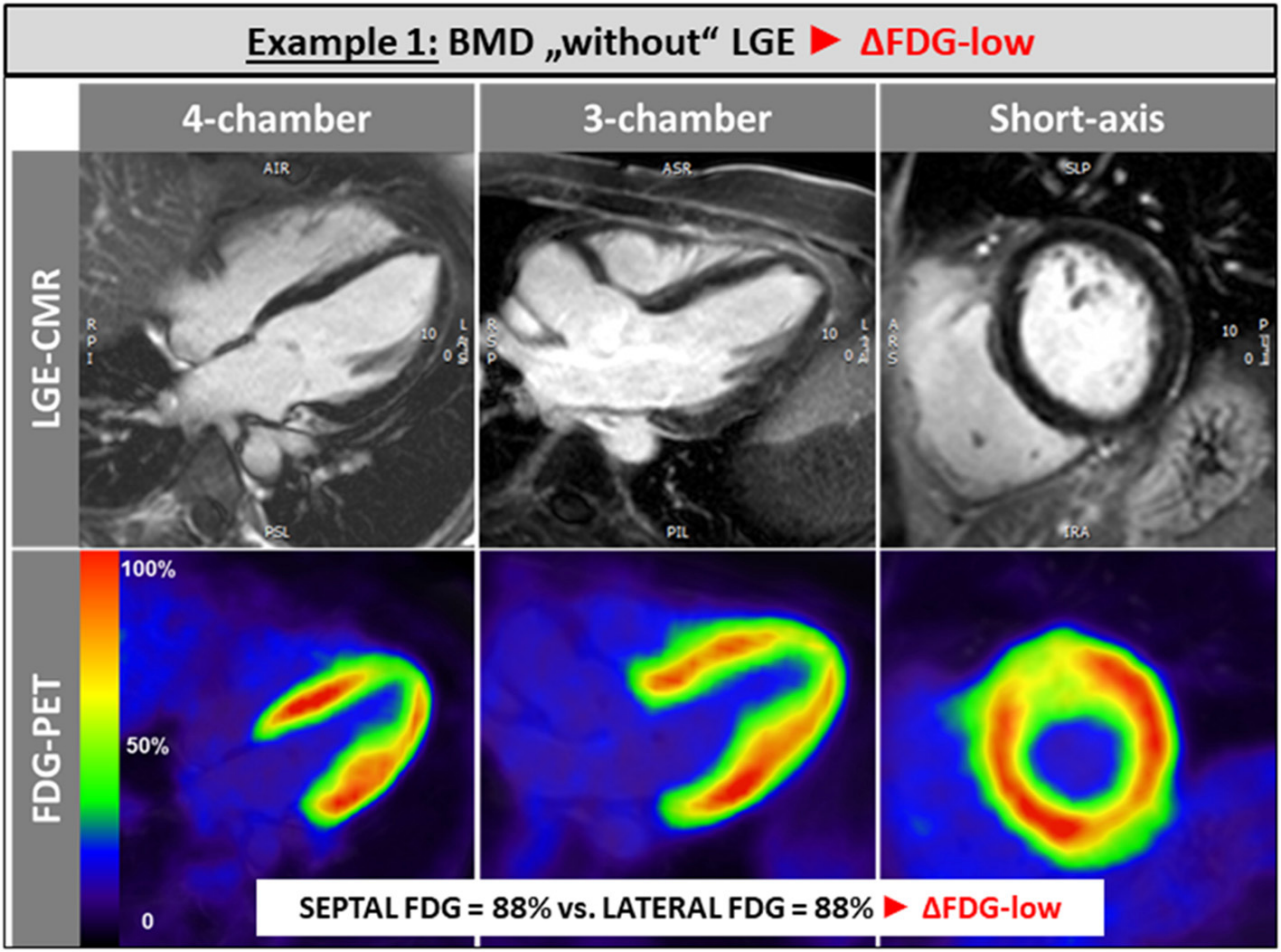
The single patient without any LGE and preserved LV and RV systolic function showed a balanced FDG uptake when septal segments were compared to lateral ones.

**Figure 2:**
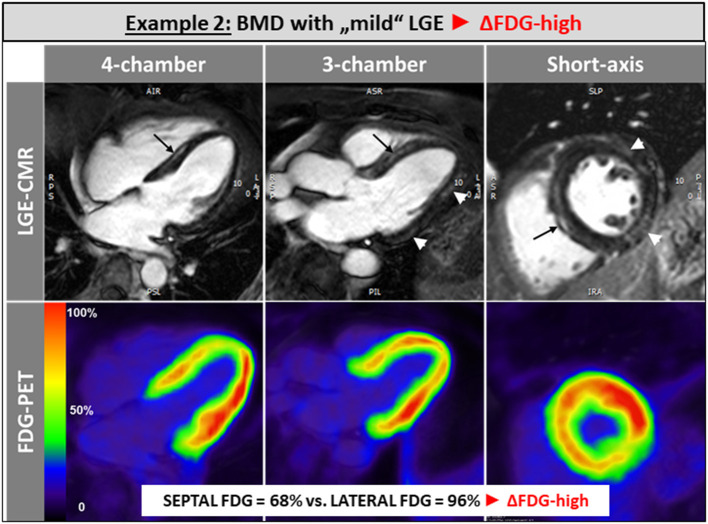
Patients in the ΔFDG-high group showed rather small areas of LGE in both the septal as well as the lateral walls without significant differences and were characterized by the highest segmental FDG uptake values in the LV lateral wall.

**Figure 3:**
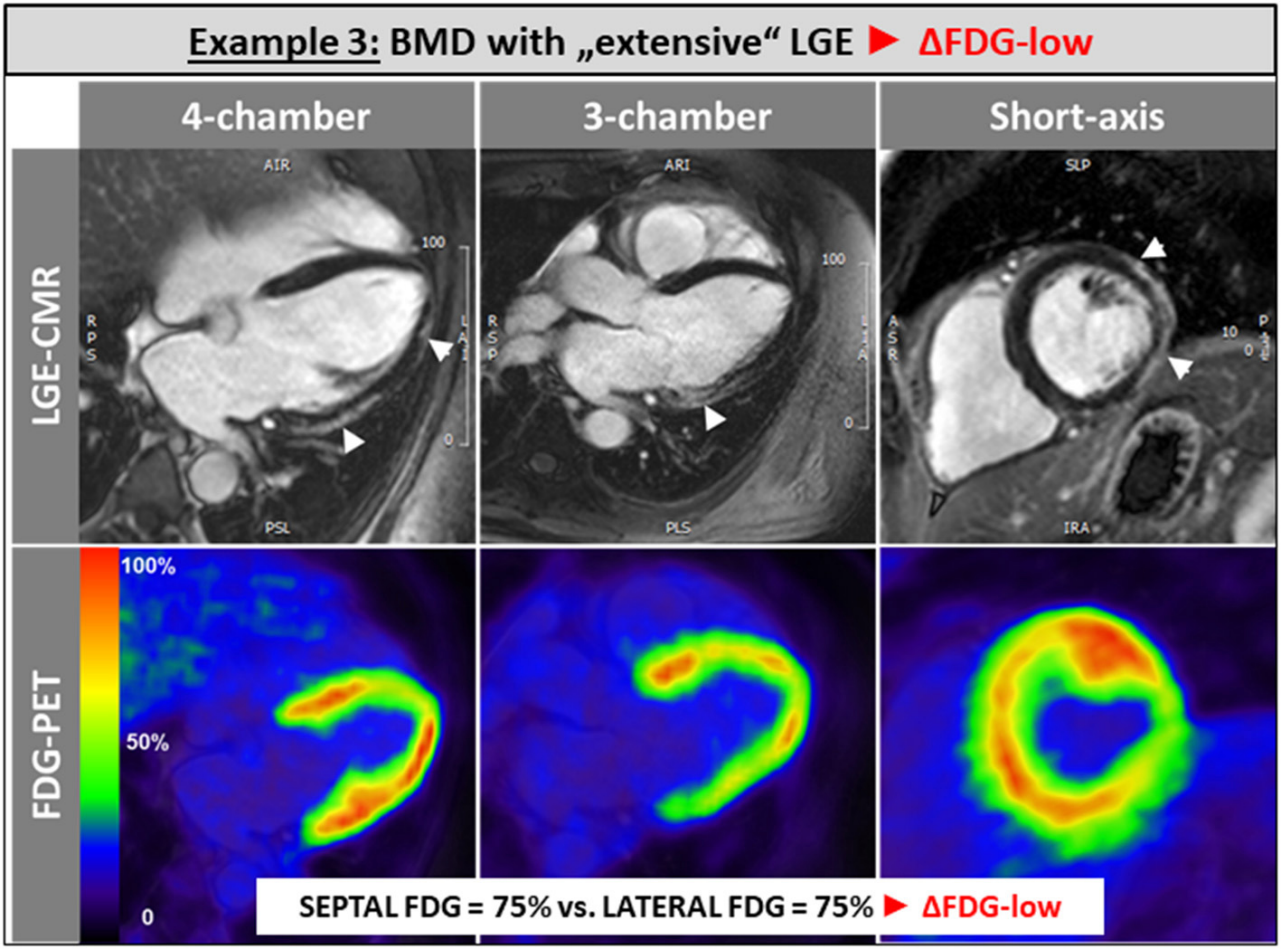
Patients in the ΔFDG-low group were characterized by rather large areas of LGE in the LV lateral wall compared to the septal wall, but showed less extensive FDG uptake in the lateral wall compared to the ΔFDG-high group.

The single patient without any LGE and preserved LV and RV systolic function showed a balanced FDG uptake when septal segments were compared to lateral ones (Figure 1). Patients in the ΔFDG-high group showed rather small areas of LGE in both the septal as well as the lateral walls without significant differences (*p* = 0.09) and were characterized by the highest segmental FDG uptake values in the LV lateral wall Table 2; Figure 2. In contrast, patients in the ΔFDG-low group were characterized by rather large areas of LGE in the LV lateral wall compared to the septal wall (*p* < 0.004), but showed less extensive FDG uptake in the lateral wall compared to the ΔFDG-high group Table 2; Figure 3.

## Discussion

To the best of our knowledge, this is the first clinical study that uses simultaneous PET-CMR imaging in patients with BMD in order to assess both metabolic (FDG-PET) and structural myocardial changes (LGE-CMR) at the same time in the same cardiac regions. Based on the presented preliminary data, we hypothesize that (a) segmental FDG uptake (reflecting myocardial metabolic activity) is higher in the LV lateral free wall (compared to the septal wall) of BMD patients possibly due to a higher segmental work load, however, only as long as myocardial scarring is limited, and (b) extensive and ongoing myocardial scarring in the LV free wall results in equalization of lateral vs. septal wall FDG uptake by a “relative” decrease in lateral wall FDG uptake due to a decreasing number of “viable” cardiomyocytes whilst growing scars in this region.

Measurement of FDG-uptake—reflecting myocardial glucose metabolism—can be influenced by different patient-specific as well as procedural factors. This important issue was recently addressed in depth by Minamimoto et al. (11) nicely illustrating that “major factors affecting myocardial glucose metabolism include sex differences, aging, obesity, and diabetic mellitus” and that “metabolic change also occurs in pathological states such as obesity, diabetic mellitus, and non-ischemic cardiomyopathy.” Moreover, these authors mentioned that “the physiological FDG-uptake … will be poorly-reproducible in the following PET examination” (!). These issues are important limitations of all FDG-PET studies and do not only concern our present study …

The presence of LGE in BMD patients is caused by progressive myocardial fibrosis as a consequence of ongoing cardiomyocyte cell death due to genetic dystrophin-deficiency. Consequently, naturally occurring LGE does not disappear in BMD patients and is rather extending in the further disease course with a continuous shift in the pattern from subepicardial to transmural. Noteworthy, this process of progressive myocardial fibrosis starts in the LV lateral free wall and extends to the septal wall segments during the natural disease course—but is always more pronounced in the LV lateral free wall.

As explained previously, myocardial fibrosis that can be depicted by LGE-imaging preferentially located in the LV free lateral wall of BMD patients is likely (a) due to increased mechanical stress in this region and (b) preceded by adaptive metabolic alterations that occur prior to final cell death. The detailed molecular pathomechanism leading to progressive cardiac contractile dysfunction in BMD patients is still not known. Early alterations in cell metabolism and signal transduction were suggested based on preclinical studies in dystrophin deficient animal models (12). Moreover, excessive intracellular calcium signaling and reactive oxygen species (ROS) generation with breakdown of the mitochondrial membrane potential were described in *in vitro* studies and may constitute the link between the initial sarcolemmal injury due to dystrophin deficiency and mitochondrial dysfunctions (12, 13). In addition, it was demonstrated that even intact dystrophin deficient cardiomyocytes have reduced compliance and increased susceptibility to stretch mediated calcium overload, which in turn lead to cardiomyocyte contracture and cell death. Hence, the fragility of the cell membrane caused by deficient sarcolemmal dystrophin may predispose cardiomyocytes to “metabolic” alterations, which in turn may be enhanced by an excessive susceptibility to mechanical stress. Therefore, it is not surprising that segmental FDG uptake is higher in the LV lateral free wall (compared to the septal wall) of BMD patients—as long as myocardial scarring in these segments is limited.

Future studies with larger sample size need to confirm the present hypothesis-generating preliminary results (and are currently performed at least at our center) using simultaneous PET-CMR imaging. Obviously, a “relatively” increased segmental FDG uptake may reflect “aggravated,” however, “reversible” metabolic changes in the myocardium (caused by e.g. disease-specific mechanical stress) and thereby precede irreversible cell death with occurrence of replacement fibrosis. Hence, FDG-PET findings—in addition to simultaneous LGE-CMR findings—may help to detect very early cardiac disease stages and thereby allow to timely start appropriate therapy and to carefully monitor cardiac disease stage.

### Limitations

Obviously, the study group was rather small and cautious interpretation of the present data and results is required. However, BMD is an orphan disease and both recruitment of patients as well as conduct of PET-CMR studies are challenging in BMD patients. Noteworthy, this is the first PET-CMR study that was performed in BMD patients so far. Second, myocardial FDG uptake is prone to variations that do not always reflect pathological changes in metabolism (e.g. in case of suboptimal oral glucose loading to effect myocardial carbohydrate uptake). We cannot rule out some “physiological” differences between septal and lateral wall FDG-uptake, but we do not believe that those “physiological” effects may explain the substantial differences observed in the present study. Finally, we have chosen a simplified and targeted approach regarding analysis of FDG-PET data in order to allow a straightforward and categorical comparison of LV lateral vs. septal wall segments—without getting lost in too complex analyses that would not be appropriate in our small study group. It was decided against full quantitation of myocardial glucose utilization since this would require a lengthy glucose clamp procedure (14) considered too time consuming in addition to the already lengthy PET-CMR imaging procedure for this patient group with the risk of early scan termination.

## Conclusion

Segmental FDG uptake—reflecting myocardial metabolic activity—is higher in the LV free wall of BMD patients—possibly due to a higher segmental work load. However, segmental metabolic activity seems to be dependent on and limited by the respective segmental extent of myocardial fibrosis as depicted by LGE-imaging.

## Data Availability Statement

The original contributions presented in the study are included in the article/supplementary material, further inquiries can be directed to the corresponding author.

## Ethics Statement

The study protocol complies with the Declaration of Helsinki and was approved by the Local Ethics Committee (Landesärztekammer Westfalen-Lippe, Reg. No. 2017-175-f-S) and the Federal Office for Radiation Protection (BfS). Written informed consent was obtained from every patient. The patients/participants provided their written informed consent to participate in this study.

## Author Contributions

VV participated in the CMR and PET exams, performed major analyses of the PET data, and critically reviewed the manuscript. FB participated in the design of this study, performed PET analyses, and critically reviewed the manuscript. AF and SD participated in the CMR exams and in the analysis of the CMR data. BC and PK participated in the analysis of clinical data and critically reviewed the manuscript. LS supported to develop the theoretical framework, participated in the analysis of PET data, and helped shape the research and manuscript. AY designed the project, provided the main conceptual idea, supervised the work, provided critical feedback and helped shape the research, analysis, and manuscript. All authors contributed to the article and approved the submitted version.

## Funding

This study was funded by a grant from the German Research Foundation (DFG) to LS (STE 1924/2-1) and AY (YI 127/5-1).

## Conflict of Interest

The authors declare that the research was conducted in the absence of any commercial or financial relationships that could be construed as a potential conflict of interest.

## Publisher's Note

All claims expressed in this article are solely those of the authors and do not necessarily represent those of their affiliated organizations, or those of the publisher, the editors and the reviewers. Any product that may be evaluated in this article, or claim that may be made by its manufacturer, is not guaranteed or endorsed by the publisher.
